# Idiopathic normal pressure hydrocephalus diagnosis: Quantitative and qualitative score predicting outcome of extended lumbar drainage

**DOI:** 10.1016/j.heliyon.2024.e31004

**Published:** 2024-05-10

**Authors:** Roberta Laera, Maria Luisa Gorgoglione, Antonello Curcio, Giuseppina Marzano, Gerardo Caruso, Mariella Caffo, Antonino Germanò

**Affiliations:** Department of Biomedical and Dental Sciences and Morphofunctional Imaging, Neurosurgical Clinic, University of Messina, Messina, Italy

## Abstract

• INPH may mimic other disorders among elderly such as Parkinson's and Alzheimer's disease.
• A systematic and established approach for diagnosis is required due to the fact that iNPH prevalence is increasing as a result of the rapidly aging population.
• When it comes to identifying individuals who may benefit from ELD and have suspected iNPH, qualitative cognition tests are more accurate than quantitative neurocognitive tests.

## Graphical abstract

**Figure:**
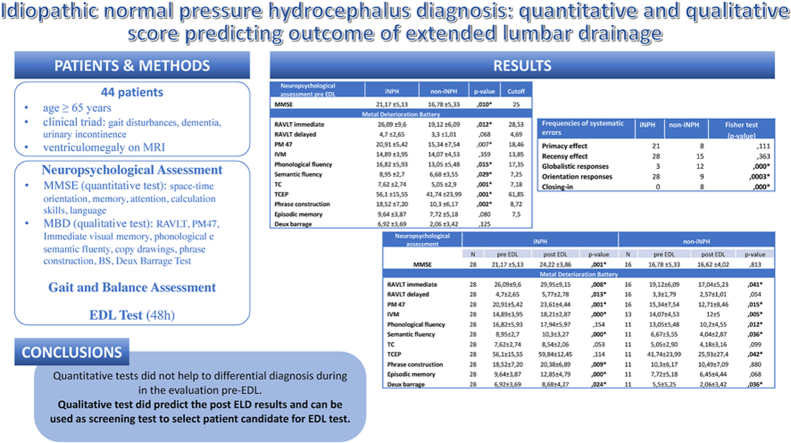

## 1 Introduction

Idiopathic normal pressure hydrocephalus (iNPH) is a neurologic disorder first described by Hakim and Adams in 1965 with a case series of patients presenting the characteristic triadic syndrome (gait disturbances, cognitive impairment and urinary incontinence) along with ventriculomegaly and cerebrospinal fluid (CSF) pressure within the normal range [1]. This condition was defined as idiopathic NPH due to its unknown origin contrary to secondary NPH caused by other neurological injury (e.g., subarachnoid or cerebral haemorrhage, meningitis). The cause of iNPH has not been fully investigated although it is not a rare clinical entity. The prevalence is growing due to rapidly aging society and has been estimated to be 10–22 per 100 000 overall, with 1.30 % in those aged ≥65 years and 5.9 % in those aged ≥80 years [2]. It manifests after the 6th decade with neuro-radiological typical findings (ventriculomegaly, sylvian fissure dilation, narrowing of the high convexity/midline subarachnoid spaces, steepening of callosal angle) [3] associated with the clinical Hakim-Adam's triad. Treatment by placement of ventriculo-peritoneal shunt (VPS) and ventriculo-atrial shunt (VAS) is successful in symptoms regression in more than 80 % of the patients [4].

To date, several tests are used for the diagnosis of iNPH in order to predict a favourable outcome following shunt placement. Those tests include both motor tests and neuropsychological assessment aimed at identifying a typical pattern of iNPH patients that can be distinguishable from other forms of dementia. iNPH grading scale (iNPHGS) [5] is a subjective scale that evaluates gait disturbance, cognitive impairment, and urinary incontinence on a scale from 0 to 4, adapting the scale to patients with a wide range of symptom severity. The total score can be used as an index, together with the evaluation points for each of the three conditions. The iNPHGS is used along with other objective and quantitative evaluation methods as the timed up & go test (TUG) [6] a valid test for evaluating walking ability in which the time required to pass from sitting to standing position and to walk forward 3 m and return to the seated position with no physical assistance is calculated. In this test if the patient was at risk of falling, the examiner followed him a half-step behind to prevent a fall without affecting the patients’ walking pace.

iNPH cognitive impairments have been described as fronto-subcortical dementia [7,8], but the term is reductive as it does not fully describe the different clinical pictures observed [9], (<b>[10]).</b> In the early stages, the cognitive profile is characterized mainly by impairments of attention, psychomotor speed and memory. Memory impairment is characterized by difficulty acquiring new information and retrieving it. Patients with more advanced iNPH show overall cognitive deteriorate, with specific alterations in executive functions, memory, working memory, information processing speed, attention, memory, visuospatial and visuo-constructional skills, psychomotor slowing and mood symptoms, especially apathy. However, iNPH symptoms are nonspecific and frequently found in other neurological disorders. The characteristics of cognitive decline in iNPH patients can be various: it can be similar to normal aging, or it manifest itself as progressive dementia with impaired gait comparable to Alzheimer's (AD) or Parkinson's disease [11] -(<b>[12]).</b> The diagnosis and criteria used to determine which patients to treat are still a controversial topic. Specific diagnostic criteria are needed to define sistematic neuropsychological markers and iNPH specific profile to achieve early diagnosis. For cognitive assessment, Mini-Mental State Examination (MMSE), the Wechsler Adult Intelligence Scale-III digit symbol coding and symbol search tasks and the Frontal Assessment Battery are recommended. The Rivermead Behavioral Memory Test, a parallel test, is a recommended memory test. Improving diagnostic procedure is needed to identify the appropriate candidates for surgery compared to patients with neurological disorders similar to iNPH, but who would not obtain advantages from the surgical procedure. Literature focus is on the analysis of quantitative differences between different cognitive domains and between pre and post CSF drainage test. The aim of his study is to examine the qualitative differences between iNPH and other neuropsychological disease to identify and differentiate the possible neuropsychological markers as an aid tool in the diagnostic procedure.

## 2 Materials and methods

### 2.1 Participants

We retrospectively analyzed data from a series of consecutive patients with suspected iNPH admitted to the Neurosurgery Unit of the University Hospital “G. Martino” of Messina, from November 2018 to November 2022. Inclusion criteria were age ≥65 years, clinical triad (gait disturbances, dementia, and urinary incontinence), ventriculomegaly on magnetic resonance imaging (MRI). Age, gender, years of education were collected (Table 1). The exclusion criterion was the presence of ventriculomegaly secondary to other pathological conditions. To discriminate patients with iNPH, our center's protocol provides a multiparametric evaluation including motor and neurocognitive assessment before and after placement of external lumbar drainage (ELD). All patients underwent neuropsychological assessment with MMSE and Mental Deterioration Battery (MDB) and they were videotaped for gait and balance analysis prior to and 2 h following ELD. ELD lasts 48 h. Two patients did not undergo motor tests due to bedridden. Based on the improvement of cognitive and motor performances post-ELD, patients were divided into two subgroups, iNPH and non-iNPH patients. 10 patients of iNPH group were undergone to surgical treatment (ventriculo-peritoneal/atrial shunting with a programmable valve). Informed consent was obtained for all patients.

**Table 1:** 44 patients included in the study. The table show age (≥65 years), gender, years of education (YoE), clinical triad (cognitive disfunction, gait disturbances and urinary incontinence). Motor and NPS tests were performed. Improvement column shows the NPS evaluation after ELD: (−) no improvement; (+) not significant improvement; (++) significant improvement. X: present/performed; NE: not executable; VPS: ventriculo-peritoneal shunt; VAS: ventriculo-atrial shunt.

Patients	Age	Gender	YoE	Clinical triad	Motor test	NPS test	Improvement	Surgery
1	67	M	5	X	X	X	–	
2	74	M	5	X	X	X	++	VPS
3	74	M	8	X	X	X	+	
4	78	F	18	X	X	X	–	
5	80	M	8	X	X	X	+	VPS
6	80	M	18	X	X	X	++	VPS
7	69	M	5	X	X	X	++	VAS
8	80	M	18	X	NE	X	++	
9	68	M	13	X	X	X	+	VPS
10	75	M	8	X	X	X	+	
11	72	M	13	X	X	X	–	
12	71	M	2	X	X	X	+	VPS
13	69	F	8	X	X	X	++	
14	75	F	8	X	X	X	++	
15	60	F	13	X	X	X	–	
16	72	F	18	X	X	X	+	
17	71	M	5	X	X	X	++	VPS
18	69	M	13	X	X	X	+	
19	63	M	8	X	X	X	+	VPS
20	83	M	5	X	X	X	++	
21	71	F	5	X	X	X	+	
22	73	F	18	X	X	X	–	
23	69	M	13	X	X	X	++	VPS
24	81	M	5	X	X	X	++	VPS
25	63	M	13	X	X	X	++	
26	64	F	18	X	NE	X	–	
27	67	M	8	X	X	X	–	
28	67	F	5	X	X	X	–	
29	49	F	8	X	X	X	–	
30	62	F	13	X	X	X	++	
31	68	F	13	X	X	X	++	
32	68	M	8	X	X	X	++	
33	72	M	8	X	X	X	–	
34	68	M	8	X	X	X	–	
35	78	M	8	X	X	X	++	
36	78	M	18	X	X	X	++	
37	75	F	5	X	X	X	+	
38	78	F	5	X	X	X	–	
39	68	F	13	X	X	X	–	
40	84	M	18	X	X	X	–	
41	74	F	4	X	X	X	+	
42	81	M	13	X	X	X	–	
43	67	F	13	X	X	X	+	
44	75	F	5	X	X	X	–	

### 2.2 Neuropsychological assessment

Our Centre protocol for the evaluation of cognitive impairment includes several tests widely used in the neuropsychological community, hereby listed.

• The MMSE [13] is a quantitative test that investigates that space-time orientation, memory, attention, calculation skills, language, in the sub-components comprehension, repetition, naming, reading and writing and praxic-constructive abilities. A score of 23 on 30 or lower is indicative of cognitive impairment. The raw score, whose cutoff is 25, is corrected for age and education.
• The MBD test [14] consists of 8 sub-tests, verbal and non-verbal, which have proven to be sensitive and valid in detecting the presence of cognitive impairment in different areas. It includes:
1) Rey Auditory Verbal Learning Test (RAVLT), for assessing immediate and deferred learning of new information. It is administered by reading the list of words to the patient, at the end of the reading, the patient is asked to repeat as many of the words just heard as possible in any order. This procedure is used, with the same list of words five consecutive times, recording each time different elements not only quantitative but also qualitative (immediate recall). After an interval of 15 min, during which visuo-spatial tests are performed, the patient is asked to remember (without the list being re-proposed by the examiner) as many words as possible from the list (deferred recall);
2) Raven's Colored Progressive Matrices, PM 47 for the assessment of logical-deductive intellectual abilities. This test studies the entire range of intellectual functions and, requiring minimal verbal and motor expressions, is particularly suitable for studying normal or pathological ageing. The test consists of 36 matrices divided into 3 sets (A-AB-B), 12 items each. Each item requires you to identify, among 6 alternatives, the piece that completes the matrix. The test is administered without time limits. One point is awarded for each correct answer. The raw score, given by the sum of the correct answers, is corrected for age and education. The cutoff for normality purposes is equal to 18,96;
3) Immediate Visual Memory Test, for evaluating the visuo-spatial working memory at a visual stimulus (PM 47 tiles). It consists of 22 items made up of single cards that the subject must memorize in 3 s, then the second table is presented which contains 4 cards vertically aligned, including the one just presented that the patient must recognize. One point is awarded for each correct answer. The raw score is adjusted for age and education and the cutoff is 13,85;
4) Phonological Fluency Test, for the assessment of access and lexical retrieval. Subjects are asked to say as quickly as possible the largest number of words that come to mind and begin with a specific letter (F; A; S). the required time is 1 min. One point is awarded for each correct word. The raw score, given by the sum of the words produced for the three categories, is corrected for age and education. The cutoff for normality purposes is 17,35;
5) Semantic Fluency Test, for evaluating the extension and usability of the vocabulary. Subjects are asked to say as quickly as possible the largest number of words that come to mind from a specific category of meaning (colors; animals; fruit; city). The time required for each category is 2 min. One point is awarded for each correct word. The raw score, given by the sum of the words produced for the four categories, is corrected for age and education. The cutoff for normality purposes is 7,25;
6) Copy Drawings, for the assessment of praxico-constructive ability and consists of freehand copying of 3 items, one two-dimensional and two three-dimensional (star; cube; house). For each item a score according to the reproduction of the perspective, the orientation of the lines, the spatial relationship. The raw score is corrected for age and education and the cutoff for normality purposes is 7,18;
7) Copy Drawings with Landmarks, for the evaluation of the praxico-constructive ability starting from programming elements. For each figure, 1 point is awarded for each completed line, incorrect graphic strokes are not counted for the purposes of the score, corrected for age and schooling. The cut-off for normality purposes is 61,85;
8) Phrases Construction, for evaluating the ability to relate lexical units in a coherent syntactic construct. Stimulus words are provided for the construction of meaningful sentences. The cutoff is 8,72 after correction for age and education.
• Babcocks Story (BS) [15], immediate and deferred recall, is used to assess episodic memory. To facilitate verbal comprehension, the test also includes mechanisms for integrating the information that is provided. This is followed by mechanisms for planning the repetition of the stored information's structure. The average of the elements remembered during the two repetitions, which can range from 0 to 28, is used to calculate the score. For purposes of normalcy, the cutoff is < −7,5.
• Deux Barrage Test [16], for the evaluation of divided attention. A sheet with 40 lines of signs is presented, among which there is one, specified at the beginning, that the subject must cross out. The score assigned is given by the combination of exact, omitted and incorrect elements.

### 2.3 Gait and balance assessment

The motor evaluation protocol used at our center includes the following tests. The 10 m walking test, which is part of the gait assessment, counts the steps and seconds required to walk 10 m at a free pace in a straight line as quickly as possible. Measurements begin from a stationary position. If the patient has a walking aid, he should use it. The test is run twice, with mean values considered. A 360° turn test was used to assess balance, and the number of steps required to complete the turn was counted. Next, the mean of the two turning trials was computed. The number of seconds in which the patient can stand up straight on one or both legs and the capacity and number of seconds it takes to sit down and stand up are calculated. Every patient performance was videotaped after consent was obtained.

### 2.4 ELD test

After motor and neurocognitive assessment, we performed ELD test, preferred over the lumbar puncture (LP) due to high positive predictive value (PPV) and high negative predictive value (NPV). Before the procedure, complete blood test including platelet count, prothrombin time and partial thromboplastin time were obtained. Anticoagulant medication must have been stopped by the patient, and all test results had to be within normal ranges. Before the drain placement, all patients received a prophylactic dose of intravenous antibiotic (cephazolin, or vancomycin if allergic to penicillin). With a sterile technique and after infiltration with lidocaine, a 8-gauge Tuohy needle was used to access the subarachnoid space in the vertebral interspace between L3-L4 and L5-S1, then 10–15 cm of a 20 gauge catheter was inserted into the subarachnoid space. After being retained for 48 h, the ELD was removed.

### 2.5 Statistical analysis

Statistical analysis was performed with SPSS (Statistical Package for the Social Science; SPSS Inc., Chicago, IL, USA). Nonparametric analysis of variance (ANOVA) was used for the descriptive analysis of neuropsychological score; Fisher test was used to compare the frequencies of systematic mistakes in iNPH and AD patients and the paired Student's t-test to evaluate the effect of ventriculoperitoneal shunting on cognitive functions in iNPH patients. p < 0.05 was considered statistically significant.

## 3 Results

**Fig. 1:**
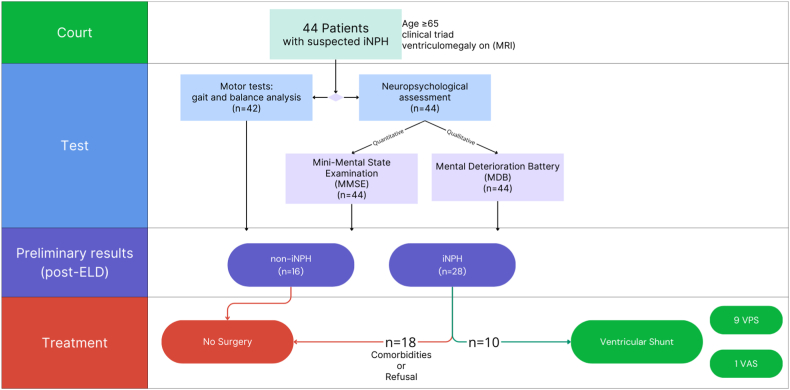
Flow-chart of selected patients.

We retrospectively analyzed data of 44 consecutive patients (26 males, 18 females; mean age = 71.6 ± 6.8). All patients underwent brain CT, MRI and neurological examination. Years of education mean was 10.9 ± 4.9y. All patients underwent neuropsychological and motor assessment prior to and 2 h following EDL (Fig. 1). Multiparameter evaluation, as previous described, suggested indication to surgery in 28 patients over the 44, that were so classified as iNPH group. VPS was performed in 9 cases and VAS in 1 case, all of them with a programmable valve set at a pressure of 140mmH2O. No postoperative complications were observed. MRI and CT scans were used for the postoperative neuroradiological evaluation six days and thirty days after the procedure, respectively. Ventricular system volume appeared slightly decreased. The remain 18 patients, diagnosed with iNPH after multiparameter assessment, did not undergo surgery due to severe comorbidities or because they refused it.

## 4 Quantitative analysis

### 4.1 Patients were assessed before and after ELD

Assessment pre-ELD of iNPH group (n. 28) shown impairment of short- and long-term memory (RAVLT), praxico-constructive abilities (TC and TCEP), phonological fluency (WFF), and divided attention (DEUX BAR- RAGE). The ELD procedure affects positively the cognitive performances of this group more than non-iNPH patients (Table 2).

**Table 2:** Neuropsychological assessment comparing iNPH vs. non-iNPH.

Neuropsychological assessment	iNPH				non-iNPH			
	N	pre EDL	post EDL	*p-value*	N	pre EDL	post EDL	*p-value*
MMSE	28	21,17 ± 5,13	24,22 ± 3,86	,001*	16	16,78 ± 5,33	16,62 ± 4,02	,813
Metal Deterioration Battery								
RAVLT immediate	28	26,09 ± 9,6	29,95 ± 9,15	,008*	16	19,12 ± 6,09	17,04 ± 5,23	,041*
RAVLT delayed	28	4,7 ± 2,65	5,77 ± 2,78	,013*	16	3,3 ± 1,79	2,57 ± 1,01	,054
PM 47	28	20,91 ± 5,42	23,61 ± 4,44	,001*	16	15,34 ± 7,54	12,71 ± 8,46	,015*
IVM	28	14,89 ± 3,95	18,21 ± 2,87	,000*	13	14,07 ± 4,53	12 ± 5	,005*
Phonological fluency	28	16,82 ± 5,93	17,94 ± 5,97	,154	11	13,05 ± 5,48	10,2 ± 4,55	,012*
Semantic fluency	28	8,95 ± 2,7	10,3 ± 3,27	,000*	11	6,67 ± 3,55	4,04 ± 2,87	,036*
TC	28	7,62 ± 2,74	8,54 ± 2,06	,053	11	5,05 ± 2,90	4,18 ± 3,16	,099
TCEP	28	56,1 ± 15,55	59,84 ± 12,45	,114	11	41,74 ± 23,99	25,93 ± 27,4	,042*
Phrase construction	28	18,52 ± 7,20	20,38 ± 6,89	,009*	11	10,3 ± 6,17	10,49 ± 7,09	,880
Episodic memory	28	9,64 ± 3,87	12,85 ± 4,79	,000*	11	7,72 ± 5,18	6,45 ± 4,44	,068
Deux barrage	28	6,92 ± 3,69	8,68 ± 4,27	,024*	11	5,5 ± 5,25	2,06 ± 3,42	,036*

ANOVA demonstrated a significant improvement in 16 patients among the 28 of iNPH group. Specifically, both short- and long-term mnemonic skills significantly improved in the episodic component, reasoning, praxico-constructive skills, word fluency and divided attention. The remaining 12 patients of iNPH group, despite an improvement post ELD, ANOVA did not demonstrate significant changes. In 16 patients ELD produced no benefits (non-iNPH group). In this group, the neuro-psychological evaluation on admission to the ward had recorded a greater impairment of global cognitive functioning compared to iNPH patients (Table 3). This finding was confirmed by ANOVA.

**Table 3:** Summery of the neuropsychological assessment in iNPH and non-iNPH groups on admission.

Neuropsychological assessment pre EDL	iNPH	non-iNPH	*p-value*	Cutoff
MMSE	21,17 ± 5,13	16,78 ± 5,33	,010*	25
Metal Deterioration Battery				
RAVLT immediate	26,09 ± 9,6	19,12 ± 6,09	,012*	28,53
RAVLT delayed	4,7 ± 2,65	3,3 ± 1,01	,068	4,69
PM 47	20,91 ± 5,42	15,34 ± 7,54	,007*	18,46
IVM	14,89 ± 3,95	14,07 ± 4,53	,359	13,85
Phonological fluency	16,82 ± 5,93	13,05 ± 5,48	,015*	17,35
Semantic fluency	8,95 ± 2,7	6,68 ± 3,55	,029*	7,25
TC	7,62 ± 2,74	5,05 ± 2,9	,001*	7,18
TCEP	56,1 ± 15,55	41,74 ± 23,99	,001*	61,85
Phrase construction	18,52 ± 7,20	10,3 ± 6,17	,002*	8,72
Episodic memory	9,64 ± 3,87	7,72 ± 5,18	,080	7,5
Deux barrage	6,92 ± 3,69	2,06 ± 3,42	,125	

### 4.2 Qualitative analysis

This retrospective study was conducted to evaluate the cognitive impairment tests utility as screening marker between iNPH and non-iNPH groups. The frequency of systematic errors differs significantly between the groups: patients identified as iNPH show a significant tendency to give orientation misdirection stimulus responses to the PM47 test, while non-iNPH group show a significant tendency to give global misresponses stimulus responses in the same task. The CT closing-in effect is another erroneous response that distinguishes iNPH patients from non-iNPH patients (Table 4).

**Table 4:** Systematic errors frequencies of qualitative tests between iNPH and non-iNPH.

Frequencies of systematic errors	iNPH	non-iNPH	Fisher test (p-value)
Primacy effect	21	8	,111
Recensy effect	28	15	,363
Globalistic responses	3	12	,000*
Orientation responses	28	9	,0003*
Closing-in	0	8	,000*

## 5 Discussion

iNPH is a complex syndrome that typically occurs in the elderly population, who frequently have other comorbidities. Several neurodegenerative diseases can mimic iNPH symptoms and, this heterogeneity makes diagnosis challenging. To date, both instrumental neuroradiological (CT scan, MRI) and neuropsychological examinations are unable to establish the correct diagnosis between iNPH and other neurodegenerative diseases. Furthermore, it has been reported in the literature how the sistematic combined use of motor and neurocognitive assessment is significantly more sensitive in identifying patients who will benefit from surgery. Our experience is in line with what above mentioned since the evaluation used for motor tests are often not very sensitive in documenting the improvement in terms of mere scores.

Neuropsychological assessment may help in differential diagnosis. Indeed, compared to other forms of dementia, iNPH is characterized by a dysfunction of executive functions and short-term memory at the cognitive level [17,18]. Conversely, AD is characterized by a global cognitive dysfunction that affects all cognitive functioning rather than individual specific functions. In this study, we aim to investigate the role of qualitative analysis of systematic errors to early detect of iNPH patients on among those who have similar symptom onsets. According to a previous work of our Center [19], qualitative analysis of percentage of errors in neuropsychological evaluation is more sensitive than quantitative evaluation in identifying patients with suspected iNPH. In literature [20], assumed the post ELD improvement as the only discriminating factor between iNPH and non-iNPH patients. ELD is a valid tool to recognize patients who will benefit from shunting since the positive test response is very reliable. However, negative tests response is not so reliably to decide ineligibility for shunting [21]. Our patients were divided into two groups (iNPH and non-iNPH) based on the improvement after the multiparametric evaluation. iNPH patients did not have a severe dysfunction of global cognitive functioning, confirming the hypothesis that iNPH is related with a decline in frontal functioning. Furthermore, iNPH group demonstrated a dissociation between the performance of phonological fluency tasks and semantic fluency tasks when compared to non-iNPH patients. Phonological fluency activates the posterior part of the left inferior frontal cortex and the left premotor area more than semantic fluency. This data would confirm the frontal damage of iNPH patients since phonological fluency is a severely compromised executive function. Short-term memory is also a cognitive ability that is impaired in iNPH. All our cases had severe impairments, but only the iNPH patients improved after ELD. However, these data lacks in predictability. They fail to differentiate between iNPH and non-iNPH patients at time zero (T0), offering only a distinction based on the response on ELD. So, we retrospective analyzed qualitative analysis of systematic errors. At T0, we distinguish two types of responses between the iNPH and non-iNPH groups in order to predict the utility of ELD. In 1935, Mayer Gross described the closing-in phenomenon (tendency to close on a model during the execution of a praxico-constructive task) as a typical neuropsychological pattern of patients with AD. This phenomenon was later identified as a highly sensitive and specific pattern of the AD in subsequent studies [22,23]. In our study, all non-iNPH patients presented this phenomenon and did not improve after ELD, demonstrating the predictive value of this type of response. The type of error in the PM47 response revealed a further neuropsychological pattern with predictive value. In our study, patients who did not improve after ELD had a significant frequency of incorrect global responses. Conversely, patients who improved significantly after ELD had a significant frequency of providing erroneous responses for stimulus orientation. Therefore, these features could guide us in early distinguish between patients who will potentially benefit from shunting and those who will not benefit. The neuropsychological patterns identified have a high specificity for distinguishing iNPH from non-iNPH symptomatology. The analysis of these phenomena could be used at the T0 for an early differential diagnosis, being also able to predict the potential usefulness in performing a ELD test. Our findings highlight the importance of a comprehensive neuropsychological evaluation in the diagnosis of this pathology, as well as the significant value of qualitative analysis in terms of predictability. This protocol could thus be a helpful tool in the early screening of elderly patients with suspected iNPH in order to determine who should undergo an invasive procedure such as the EDL.

## 6 Limitations of the study

This study is a retrospective analysis of a consecutive case series of patients admitted to our department with the diagnostic suspicion of iNPH. All patients undergone multiparameter protocol of study as routinely performed in our Center to diagnose iNPH. Data were collected from medical history and then retrospectively analyzed. Limitations of our study included a small sample size, the lack of differentiation among comorbidities (e.g., diabetes, hypertension, etc …) the presence of patients with mood disorders, and the lack of stratification for onset and severity of symptoms. This might lower the statistical power of the study given the potential influence of these factors on cognitive test results.

## 7 Conclusions

Although our protocol has had wide inclusion criteria, the result confirms that iNPH symptoms can be still confused with those of other neurodegenerative diseases. For this reason, a standardized diagnosis protocol is mandatory. Our protocol aim is to identify patients who will benefit from surgery through ELD test. Neuropsychological assessment offers a useful additional tool for differential diagnosis. We found significant improvements in neuropsychological test results after ELD in 28 of the 44 patients. Quantitative tests were not able to predict these changes and to provide differential diagnosis at T0. On the other hand, with the retrospective evaluation of the qualitative analysis of the systematic errors we found a new reading key, possibly predicting of the post ELD result. If systematically applied and interpreted, qualitative analysis of systematic errors can contribute to the early diagnosis and management of iNPH, managing to have predictive value in those patients who can benefit from ELD procedure.

## Funding

This research received no external funding

## Institutional review board statement

Not applicable.

## Informed consent statement

Not applicable.

## Data availability statement

Not applicable.

## CRediT authorship contribution statement

**Roberta Laera:** Writing – original draft, Investigation. **Maria Luisa Gorgoglione:** Writing – original draft, Formal analysis, Data curation. **Antonello Curcio:** Writing – review & editing, Software. **Giuseppina Marzano:** Validation, Data curation. **Gerardo Caruso:** Project administration, Conceptualization. **Mariella Caffo:** Visualization. **Antonino Germanò:** Project administration, Methodology.

## Declaration of competing interest

The authors declare that they have no known competing financial interests or personal relationships that could have appeared to influence the work reported in this paper.
